# Parasitology at the heart of the “One Health” concept: a longstanding alliance illustrated by cysticercosis control

**DOI:** 10.1051/parasite/2026008

**Published:** 2026-03-02

**Authors:** Jean Dupouy-Camet, Gholamreza Mowlavi, Negar Bizhani, Mohamed Gharbi, Pascal Boireau

**Affiliations:** 1 Faculté de Médecine Paris Cité 15 Rue de l’École de Médecine 75006 Paris France; 2 School of Public Health, Tehran University of Medical Sciences Pour Sina St P.O. Box 6446 Tehran 14155 Iran; 3 Center for Research of Endemic Parasites of Iran (CREPI), Tehran University of Medical Sciences Pour Sina St P.O. Box 6446 Tehran 14155 Iran; 4 Department of Biotechnology, Biotechnology Institute, Gümüşdere Kampüsü, Ankara University 06135 Keçiören/Ankara Türkiye; 5 IDEALISS ULR 7519, École vétérinaire UniLaSalle de Rouen 76130 Mont-Saint-Aignan France; 6 Jilin University, State Key Laboratory for Diagnosis and Treatment of Severe Zoonotic Infectious Diseases 5333 Xi’an Road 130062 Changchun Jilin PR China; 7 Institute of Animal Science, Chinese Academy of Agricultural Sciences 2 Yuanmingyuan West Road Beijing 100193 PR China

**Keywords:** One Health, Parasitology, Zoonoses, Cysticercosis, Multidisciplinary approach, Disease control, Public health

## Abstract

The “One Health” concept, emphasizing the interdependence of human, animal, and ecosystem health, has gained renewed global attention and institutional support from the World Health Organization, Food and Agriculture Organization of the United Nations, United Nations Environment Program, and World Organization for Animal Health. Here we underline that some principles of parasitology are embedded in this concept. As early as the 19th century, Rudolf Virchow affirmed the unity of human and veterinary medicine, a vision long practiced by parasitologists through their multidisciplinary work on zoonotic diseases. The classical “One Health” triad (humans, animals, and ecosystems) closely mirrors the complex life cycles of many parasitic zoonoses, where distinct stages circulate among hosts and ecosystems. Parasitology societies worldwide have fostered collaboration among scientists, veterinarians, physicians, and other professionals, embodying some aspects of the “One Health” approach well before its formal recognition. Using cysticercosis as an example, this article illustrates how a multisectoral, integrated framework could support effective disease control. We argue that implementing a comprehensive “One Health” strategy to combat parasitic diseases requires a systemic approach that encompasses not only veterinary and human medicine, but also ecology, the social sciences, and economics. This approach must explicitly consider research objectives related not only to human and animal health, but also to ecosystem health.

## Abstract (fr)

Le concept « Une seule santé » (*One Health*), qui souligne l’interdépendance entre la santé humaine, animale et des écosystèmes, connaît un regain d’intérêt mondial et bénéficie du soutien institutionnel de l’Organisation mondiale de la santé, de l’Organisation des Nations Unies pour l’alimentation et l’agriculture, du Programme des Nations Unies pour l’environnement et de l’Organisation mondiale de la santé animale. Toutefois, la Parasitologie intègre certains des principes de *One Health* depuis plus d’un siècle. Dès le XIX^e^ siècle, Rudolf Virchow affirmait l’unité de la médecine humaine et vétérinaire, une vision mise en pratique depuis longtemps par les parasitologues à travers leurs travaux multidisciplinaires sur les maladies zoonotiques. La triade classique d’« Une seule santé » (êtres humains, animaux, environnement) reflète étroitement les cycles biologiques complexes de nombreuses zoonoses parasitaires, au sein desquels différentes stades se transmettent entre hôtes et écosystèmes. Les sociétés de Parasitologie à travers le monde ont favorisé la collaboration entre scientifiques, vétérinaires, médecins et autres professionnels, incarnant ainsi l’approche « Une seule santé » bien avant sa reconnaissance officielle. En prenant la cysticercose comme exemple, cet article illustre comment un cadre intégré et multisectoriel pourrait soutenir un contrôle efficace des maladies. Nous soutenons que la mise en œuvre complète des stratégies « Une seule santé » contre les maladies parasitaires nécessite une perspective systémique englobant non seulement la médecine vétérinaire et humaine, mais aussi l’écologie, les sciences sociales et l’économie. Cette approche doit explicitement prendre en compte les objectifs de recherche liés non seulement à la santé humaine et animale, mais aussi à la santé des écosystèmes.

## Introduction

“One Health” has become a fashionable concept elaborated with the main goal of showing the interdependence of human medicine, veterinary medicine, and environmental care.

Parasitology, the scientific study of parasites, emerged as a distinct discipline in the late 19th century. The term “parasitology” first appeared in 1870 in the “American Naturalist,” while “parasitologist” was coined in 1862 by T. S. Cobbold (see Appendix). This timing coincides with a period of rapid advancement in both human and veterinary medicine, setting the stage for parasitology to become a bridge between these fields, a role that would later align closely with the “One Health” concept.

As discussed below, the term “One Health” is a recent creation dating from the beginning of the 21st century that was developed in the different fields of human and veterinary medicine. Kaplan *et al.* (2009) considered that “*One Health has indeed become the “Rosetta Stone” for a health-enlightening paradigm shift revolution*.” [30].

This article aims to demonstrate how parasitology has contributed to the development and operationalization of “One Health,” particularly through the study of zoonotic parasites and vector-borne diseases [30, 44]. We illustrate this dynamic using historical and contemporary examples, such as the eradication of *Taenia solium* from northern Peru and pioneering research on plague ecology in Iran. Additionally, we provide insights from generalist parasitologists who have spent decades conducting fieldwork to combat parasitic diseases and now seek to share their expertise to strengthen “One Health” strategies.

## Recent history of “One Health”

**Figure 1:**
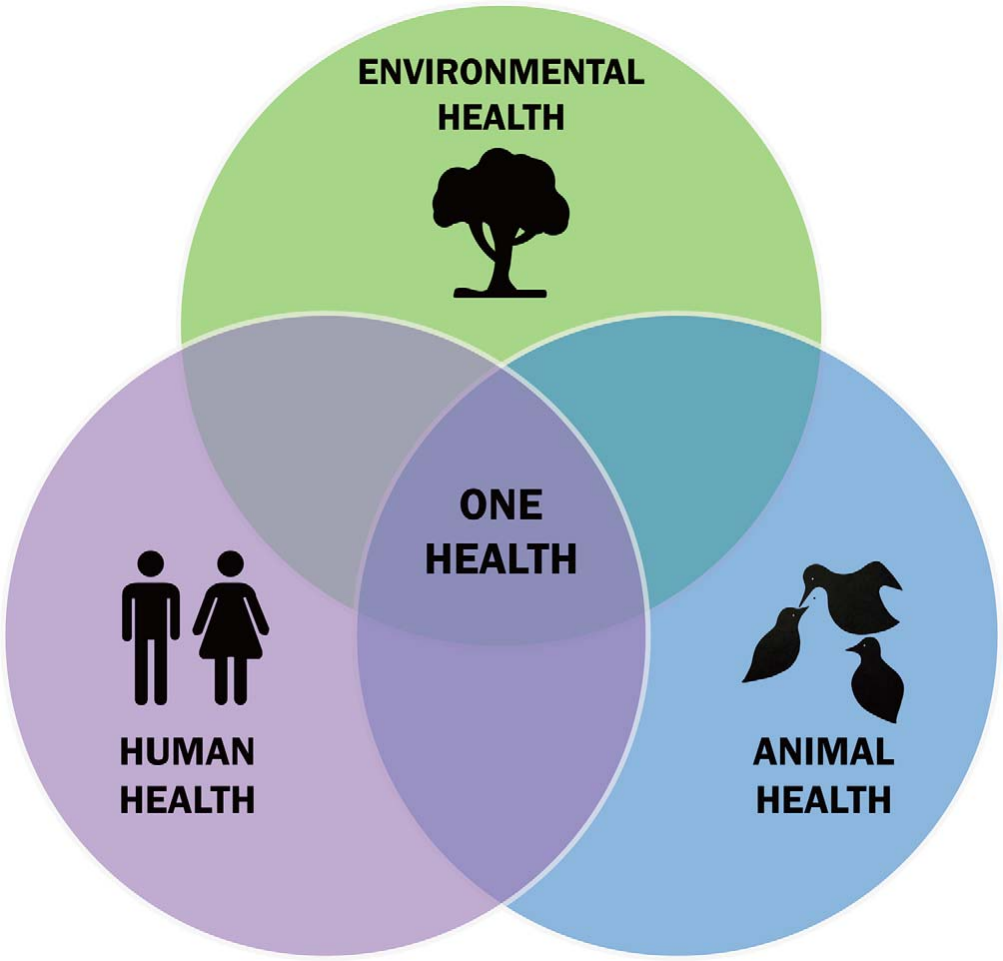
Schematic representation of the “One Health” concept. In this classic representation, “One Health” is at the center, and humans and animals are very external to the environment, which is not completely accurate. CCBY Licence.

The “One Health” concept, which emphasizes the interconnectedness between human, animal, and ecosystem health, was shaped by contributions from various pioneers and found its origin in the premises of human civilizations, which laid the groundwork for this holistic approach. The Greek physician Hippocrates emphasized, in his work “Airs, Waters, and Places,” the influence of the environment on human health. Ayurvedic or traditional Chinese medicine, the world’s oldest medical systems, stressed the balance between humans, animals, and nature [51]. Scholars of the Islamic Golden Age Medicine, like Ibn Sina (Avicenna), emphasized the role of hygiene, environmental health, and the transmission of diseases between animals and humans. In the 19th century, Rudolf Virchow (see Appendix) was considered the father of this uniqueness of medicine; it is appropriate to associate with this paradigm Sir William Osler (see Appendix), the father of modern medicine [8]. Virchow, like Osler, was enthusiastic about comparative pathology. But at the end of the 19th century and the beginning of the 20th century, the notion of a One Medicine was widely practiced in research. Louis Pasteur later bridged the worlds of fermentation, infectiology, and public health through his research on rabies and anthrax [46]. This concept of “One Medicine” was associated, in 1984, with the term “one Pathology” thanks to the veterinarian Calvin Schwabe (see Appendix), which brought together the two medicines, human and veterinary, which had become very individualized during the 20th century. The current term “One Health” was officially adopted in 2004, during a conference of the Wildlife Conservation Society [20], a non-governmental organization involved in wildlife stewardship through science and education (https://wildlife.org). This event highlighted the importance of better understanding diseases and the ecology of wildlife when it comes to dealing with the emergence of new diseases. The expression “One World, One Health” was then used to encompass both medicine and ecosystem health. Twelve recommendations (known as the Manhattan Principles, as the meeting was hosted by Rockefeller University in New York City) were made to establish a more integrated approach to the prevention of epidemic diseases and the maintenance of the integrity of ecosystems (https://www.wcs-ahead.org/manhattan_principles.html). In 2005, the “Veterinary Record” and the “British Medical Journal” published a joint issue under the title “Human and animal health: strengthening the link.” This approach, with a focus on emerging infectious diseases, was motivated by the emergence of worrying zoonotic diseases such as bovine spongiform encephalopathy, severe acute respiratory syndrome (SARS), and H5N1 influenza. The aim was to strengthen collaborations between physicians and veterinarians [20]. This concept was taken up in 2008 by FAO-WOAH-WHO (2010) under the name of “tripartite” and was promoted at the global level, particularly in low-income countries [16, 18]. These documents were then illustrated by the classical schematic representation of “One Health” with the three sectors of human, animal, and environmental health (Fig. 1).

Finally, in March 2022, the Food and Agriculture Organization of the United Nations (FAO), the World Organization for Animal Health (WOAH), the UN Environment Program (UNEP), and the World Health Organization (WHO) signed a groundbreaking agreement, known as the “quadripartite,” to strengthen cooperation “to sustainably balance and optimize the health of humans, animals, and ecosystems.” Zang *et al.* (2024) insisted upon the fact that “*good agricultural practices supported by scientific and technological advancements are essential for aligning productivity with the One Health vision, ensuring the health and resilience of all the sectors*.” [53]. Social sciences are also of importance, as noted by Estebanez & Boireau (2022), who regret that “*the questions of practices, social representations, but also of the environment are less present than the issues of human and animal medicine*.” [15].

Regarding parasitic diseases, the FAO, UNEP, and WHO published guidelines for the surveillance, prevention, and control of echinococcosis as early as 1981 [14]. Similar guidelines were later issued by the FAO, WHO, and WOAH concerning cysticercosis and trichinellosis [13, 36]. While these initiatives are often cited as early examples of a “One Health” approach, predating the actual coining of the term, they lacked a critical pillar: ecosystem health. Because these efforts focused almost exclusively on the interface between human and animal medicine without addressing environmental factors, they are more accurately described as “One Medicine” rather than true “One Health.”

## Deciphering the *Trichinella* biological cycle and “One Health”

Rudolph Virchow created, in 1855, the term “zoonosis” and elucidated the *Trichinella spiralis* life cycle in 1858. He made two particularly important observations: after infecting a dog with parasitized pork, he observed adults in the intestine and showed that, after heating the parasitized pork for 10 min, the parasite was inactivated [50]. In 1860, Friedrich von Zenker evidenced the pathogenic effect of *Trichinella*. On January 12, 1860, a 20-year-old housekeeper was hospitalized in Dresden, Germany for asthenia, fever, abdominal pain, myalgia, leg swelling, and pneumonia. She eventually died. At necropsy, von Zenker found numerous active mobile larvae in the muscles and intestinal adults, including viviparous females like those described by Virchow. *Trichinella* larvae were also found in a piece of pork consumed by the patient and kept salted [52]. The link between the parasitized pork and the disease was made. This disease, affecting humans, pork consumption, and pig farming, is the prototype of a “One Medicine” disease, as its control requires coordinated actions from physicians and veterinarians at the farm level. These works on trichinellosis led Virchow to state at the same period that “*there is no scientific barrier between veterinary and human medicine, nor should there be: the experience of one must be used for the development of the other*.” Interestingly, already in a “One medicine” approach, in 1866 two scientific missions (one French and one Austrian), each conducted by a veterinarian and a physician, traveled to Germany to learn more about trichinellosis. Both missions lasted several weeks, met the medical, scientific, and veterinary luminaries of the time in the country (Virchow, Gerlach, Fiedler, Müller, *etc.*), and formed an opinion on the disease and its prevention [10, 12, 31]. The example of trichinellosis perfectly illustrates that efficient disease control requires a coordinated effort between physicians, veterinarians, and key stakeholders, including farmers, butchers, and cooks. This 19th-century collaborative model prefigured the modern strategy for combating parasitic zoonoses through a “One Medicine” approach, where interdisciplinarity is paramount.

## Parasitologists and “One Health”

**Figure 2:**
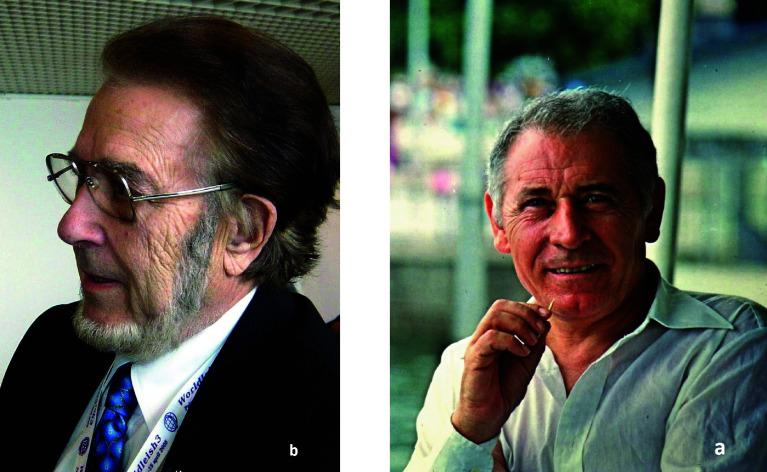
From left to right, Jean-Antoine Rioux (1925–2017) and Yves-Jean Golvan (1928–2008) (Creative Commons Attribution 4.0 International licenses).

**Figure 3:**
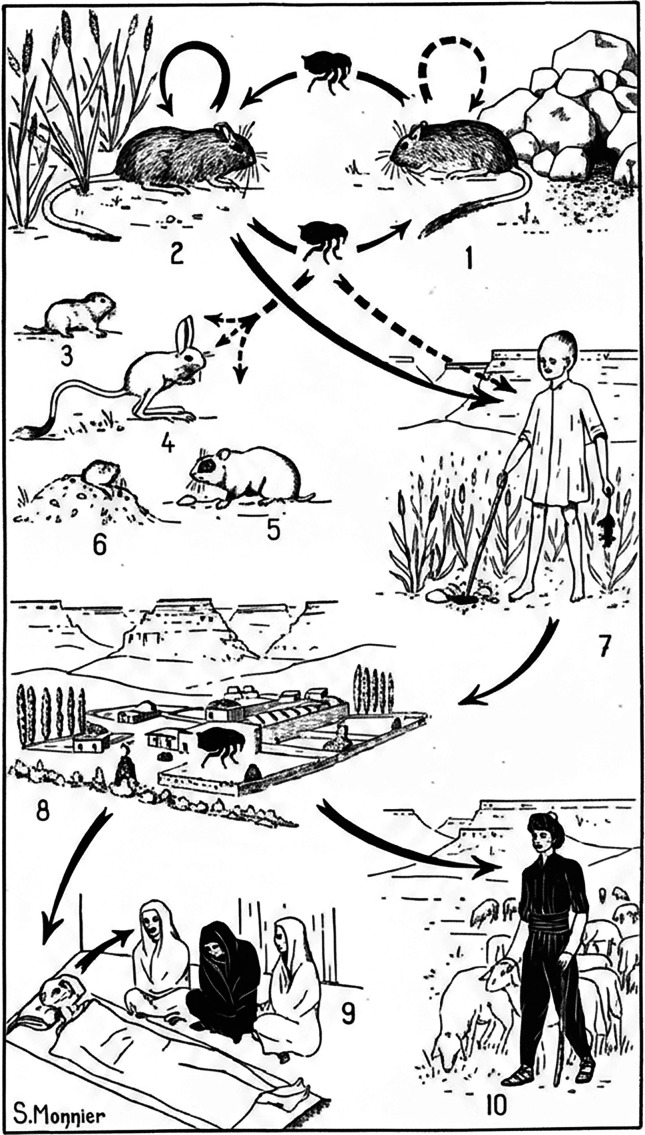
Epidemiologic cycle of the plague in Kurdistan (Golvan & Rioux, 1961): 1: *Meriones persicus*, a resistant species, in its biotope: rock gardens; 2: *Meriones vinogradovi*, a sensitive species, in its biotope: the dry wheat field on flat ground; 3: *Microtus*; 4: *Allactaga*; 5: *Mesocricetus*; 6. *Ellobius*, “vicariant reservoirs” of the plague bacillus or plague fleas; 7: Contamination of a child by handling a dead rodent; 8, 9, and 10: Village epidemic, either bubonic by the human flea or pulmonary by direct transmission. Permission granted by Société Française de Parasitologie, License: CCBY 4.0.

The discovery of parasitic life cycles, particularly those of zoonotic parasites and vector-borne diseases, required the collaboration of many scientists. The “One Health” approach, which recognizes the interconnectedness of human, animal, and ecosystem health, has roots in multiple disciplines, including veterinary medicine, ecology, epidemiology, and public health. Many parasitic diseases require such an integrated approach, as they involve human, animal, and environmental factors. Parasitologists have long been aware of the role played by environmental conditions in the spread of parasitic disease, reinforcing the need for an ecological perspective in health. The study of parasites in livestock and pets (*e.g.*, *Echinococcus* spp., *Toxocara* spp., *Trichinella* spp., *Toxoplasma gondii*, cysticercus larvae, *etc.*) exemplifies how human and animal health are interconnected. This multidisciplinary approach was used for the extensive works conducted in Iran in the 1960s by two French parasitologists to study plague epidemiology. Plague is not a parasitic disease, but a bacterial infection caused by *Yersinia pestis* and spread by fleas. At the request of Dr. M. Baltazard (see Appendix), director of the Pasteur Institute in Tehran, and of the French Ministry of Foreign Affairs, Jean-Antoine Rioux (Fig. 2a) and Yves-Jean Golvan (Fig. 2b) were asked to carry out epidemiological studies on rural plague in Kurdistan and to evaluate its relationships with the ecology of *Meriones* (gerbils), the reservoir of *Y. pestis*. Both scientists spent two years in Iran, from 1958 to 1960, and extensively studied *Meriones* populations, habitats, soil, landscape, and feedings, and published their results in a 139-page article in the *Annales de Parasitologie Humaine et Comparée* [25]. They observed that plague outbreaks significantly impacted populations of *Meriones*, suggesting that the disease functioned as a natural regulatory mechanism for these rodent communities. Their study highlighted the complex interactions between *Y. pestis*, rodent hosts, and flea vectors in the region. Their work demonstrated that plague in the Kurdish-inhabited area was not merely a sporadic human disease, but an endemic zoonosis sustained by complex ecological interactions among resistant and susceptible rodent populations and their ectoparasites. They emphasized the importance of understanding these ecological relationships to encompass the persistence and spread of the plague in natural settings. This work contributed to the broader understanding of zoonotic diseases and the ecological factors influencing their transmission. This work was the first in a long series of studies that Jean-Antoine Rioux later termed “ecoepidemiology,” aligning with early concepts that helped to develop the “One Health” approach. They concluded their paper by publishing this epidemiological cycle of the plague in which, obviously, the three sectors of the “One Health” triptych are represented (Fig. 3). Rioux and Golvan were among the first to show that *Y. pestis* could act as a natural population-regulating factor in rodent communities, shaping host dynamics and leading to periodic epizootics. Their integrated, ecology-based approach laid the groundwork for modern “eco-epidemiological” studies of the plague, and their data on host-vector relationships and environmental determinants remain foundational references not only in plague research, but also for many parasitic diseases. Jean-Antoine Rioux (see Appendix) was a renowned expert on leishmaniasis and sandflies [29]. Yves Golvan (see Appendix) was an outstanding teacher and a skillful sketcher and painter [43]. Entire generations of medical students keep a vivid memory of Golvan’s lecture course on plague epidemiology, where he brilliantly retraced all their hypotheses. Interestingly, Golvan, a physician, was also a systematician of Acanthocephala and his work on this group is more cited than his work on the plague! A physician able to work on non-medical parasites and hosts is better able to understand the interactions between human health and the environment. Nowadays, parasitologists widely advocate for a “One Medicine” framework to control zoonotic parasitosis, a strategy that serves as a vital steppingstone toward a comprehensive “One Health” perspective. Toxoplasmosis serves as a paradigmatic example of this synergy; since the causative agent, *Toxoplasma gondii*, infects all warm-blooded animals including humans, its management calls for an integrated medical and veterinary response. To reduce the disease burden of toxoplasmosis in humans, interventions are needed in the animal reservoirs, requiring close collaboration between both the human and veterinary medical sectors [11]. Recently, Gharbi and Giraudoux (2024) considered that controlling cystic echinococcosis in Tunisia necessitated the use of the “One Health” framework to improve the effectiveness of future programs. In this framework, they recommended targeting in a single program three major zoonotic diseases where dogs play a significant role: rabies, leishmaniasis, and cystic echinococcosis [19]. However, even in this attempt, the notion of ecosystem health was poorly addressed if not at all. Liu *et al.* (2025) also pointed out in a recent review on foodborne parasites that “*a thorough comprehension of the biodiversity of foodborne zoonotic parasites in China was crucial for formulating efficacious public health strategies*.” [32]. Many other examples could be given, such as cysticercosis, which will be detailed below.

## From parasitic biological cycles to the “One Health” representation

**Figure 4:**
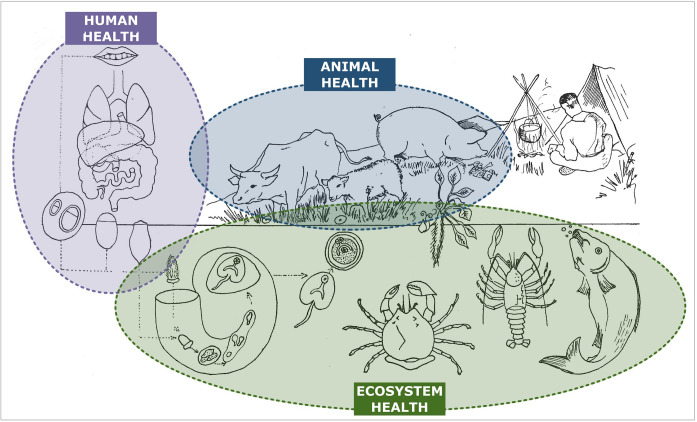
Synthetic diagram showing the “epidemiology of distomatosis” published by Harant (1939). Many distinct types of flukes are summarized here (*Fasciola, Paragonimus*, *Clonorchis*, *etc.*). The colored circles were drawn by the authors of the present paper and clearly indicate the three sectors of “One Health.”

**Figure 5:**
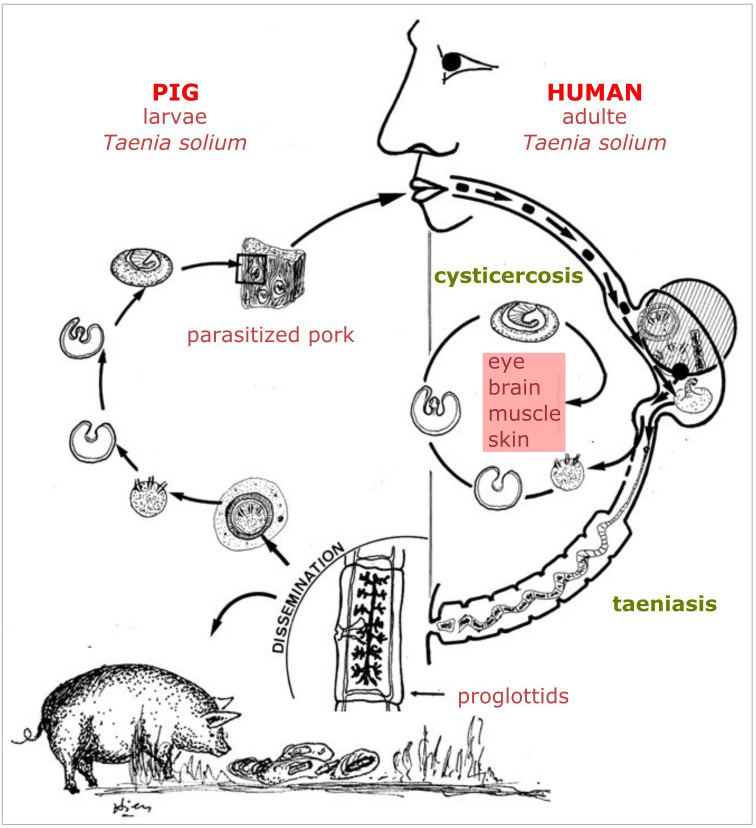
One example of a biological life cycle drawn by Tran Vinh Hien around 1970. The parasite (here *Taenia solium*) circulates between pigs, humans, and the environment (collection Jean Dupouy-Camet).

The classical schematic representation of “One Health,” with the human, animal, and environment sectors connected with arrows, is like many life cycles of zoonotic parasites, where different parasitic stages are circulating between human beings, animals, and the environment. In a recent paper, Morley (2025) noticed that the diagrammatic representations of life cycles “*began to emerge in the last decade of the 19th century, initially arising from simple charts that demonstrated the different parasite morphological life stages. Presumably intended as a more dynamic way of portraying sequential development (parasite-centric’ illustrations), they were originally restricted to representing the chronological life history stages of protist species. It was only towards the end of the second decade of the 20th century that life cycles began to be visualized for helminths, where a greater emphasis was placed on illustrating the different host species or host organ/niches required for each stage of the life cycle (‘host-centric’ illustrations’)*” [34]. As early as 1918, Chandler illustrated the trematode chapter of his book “Animal Parasites and Human Disease” with a diagram showing the distinct stages of *Fasciola hepatica* in their environment (aquatic plants and water) and within the lymnaeid intermediate host [9]. The definitive hosts were not depicted. Interestingly, the title of his book can be seen as an early foreshadowing of the “One Health” approach. However, the target was to eliminate human and domestic animal parasites, but the notion of ecosystem health was not addressed. The oldest representation of a life cycle (representing all hosts implicated) we could find was in a small 200-page booklet (“Petit Précis”) by Hervé Harant (see Appendix) and entitled “Parasitologie médicale.” On page 118 of this book published in 1939, there is a “Synthetic diagram recalling the epidemiology of distomatosis” (Fig. 4). All the sectors between which the parasites circulate were mentioned, but they were not linked by arrows [28]. After World War II, Gerhard Piekarski, a German medical parasitologist at Bonn University, graphically detailed the life cycles of the most important parasites in his “Medical Parasitology in Plates,” published in 1962, to show the circulation of parasites between different sectors: humans, animals, and the environment [40]. Arrows illustrated circulation of the parasite. Around the 1970s, most textbooks of parasitology used these schematic representations of the biological cycles of parasites, as the educational qualities of these representations were obvious [26, 37]. Tran Vinh Hien (see Appendix), working at Cochin University Hospital in Paris, illustrated in the 1970s the handout for medical students with such schematic cycles (Fig. 5).

Parasites often rely on multiple environments to complete their life cycles or proliferate. While other infectious agents, such as bacterial zoonoses or arboviruses, may also transition between environments, their life cycles tend to be less intricate. For parasites, only a specific stage of their life cycle involves passage through a particular host, be it animal, human, or environmental. Consequently, any changes to the environment directly impact their development and spread. Understanding these life cycles in detail is crucial for developing effective control strategies. It is possible to illustrate this topic with a success story: the control of cysticercosis in Peru.

## An example of a “One Health approach” to eradicate pig cysticercosis

*Taenia solium,* with its cysticercus larva, is a parasitic cestode circulating between pigs and humans. Pollution of the animal’s environment by human feces carrying eggs is the cause of pig infestation. Humans are infested by the consumption of raw or rare pork containing live cysticercus larvae. However, the parasitic cycles have variations: *Taenia solium* eggs released by humans can also provoke cysticercosis in another human or a child directly, without passing through the intermediate host. This cycle does not allow the larvae hatched from the eggs to develop into an adult. In this case, the larvae will migrate to muscles, skin, and different organs, such as the brain or the eyes. Neurocysticercosis develops when larvae lodge in the central nervous system, leading to neurological manifestations ranging from mild symptoms (*e.g.*, headache, dizziness) to seizures, intracranial hypertension, and dementia. Importantly, *T. solium* has no animal reservoir other than domestic and feral pigs. Veterinary control at the slaughterhouse is quite effective in identifying parasitized animals and removing them from consumption. An antiparasitic vaccine exists in pigs, and treatment with albendazole in humans is highly effective. Finally, there are specific and sensitive serological tests for diagnosis (ELISA, Western blot). The disappearance of *T. solium* from Europe and the United States was due to improved sanitation, regulated meat inspection, and industrialized pig farming, which disrupted the human–pig transmission cycle. Economic development and better hygiene made ongoing transmission rare. However, this parasitic disease is still a huge public health concern in some parts of Asia, Africa, and South America, where it is a leading cause of epilepsy. It is therefore possible to control and eradicate this parasite by simultaneously improving the environment and pig farming. To do this, it is necessary to combine strategies and implement a plan that meets the “One Health” criteria. Hector Garcia, in the early 2000s [17, 21], proposed a comprehensive plan to control cysticercosis in northern Peru, which had a high prevalence of *T. solium* infestation. In this plan, social well-being was a target with the desire not to destabilize the village economy based on small pig farms. Educational measures for the youngest inhabitants were implemented. Information on the disease was provided at school to ensure that the action was continued over time. Teachers were mobilized with appropriate leaflets and presentations. The human population was given systematic treatment against taeniasis. Physicians were also encouraged to prescribe treatments with financial assistance from the project. Village mayors were required and funded to provide safe drinking water and remove latrines in contact with pig farms. Veterinarians were mobilized at the slaughterhouse to check carcasses and eliminate positive animals from the food chain. Farms with positive carcasses were subjected to serological surveillance with the elimination of positive pigs. Newly introduced pigs were vaccinated. This global strategy based on a “One Health” approach involving multiple institutions, different health or education professionals, officials, and breeders, enabled the local eradication of this parasitosis with a certain cost borne, among others, by the Bill & Melinda Gates Foundation, Wellcome Trust, and the National Institutes of Health [17]. The eradication of cysticercosis based on a global assessment of the strategy is therefore possible, but at a certain financial cost and political and public will. Drastic methods, such as the off-ground breeding of pigs and their concentration, could also eradicate cysticercosis, but would also destroy a huge part of the local economy based on family pig farms and disorganize the territory’s social fabric in some localities. Finally, social upheavals, such as economic crises, conflict, or the collapse of public services, could trigger a resurgence of cysticercosis. This is especially likely in vulnerable communities where sanitation, food safety, and veterinary oversight deteriorate, particularly if the parasite persists undetected at sub-clinical levels. Effective prevention therefore depends on maintaining these systems even during periods of crisis.

## “One Health” and paleoparasitology

The successful control of cysticercosis in Peru illustrates how coordinated, multisectoral interventions can disrupt a complex zoonotic transmission cycle. While such programs operate in the present, they also echo a much longer history of interactions between humans, animals, and their environments. Bruschi *et al.* (2006) reported an ancient case of cysticercosis that was discovered in an Egyptian mummy of a young woman who lived in the late Ptolemaic period. Microscopic examination of sections of this lesion revealed a characteristic cystic structure. Immunohistochemical testing with serum from a *T. solium*-infected human confirmed the identity of the cyst. This finding confirms that, in Hellenistic Egypt, the farming of swine, along with man as an intermediate host of this parasite, was present [6]. Years before, in 1910, Ruffer (see Appendix), describing *Schistosoma* eggs in an ancient Egyptian mummy, laid the foundations of paleoparasitology. Paleoparasitology bridges the past and present, offering a unique perspective on infectious diseases that helps “One Health” policies to address today’s challenges. This historical context enriches surveillance programs, strengthens zoonotic disease prevention, and provides a valuable long-term view of human-animal-environment health interactions. Interestingly, the team led by one of the present authors (G. Mowlavi; TUMS) explicitly applied the “One Health” concept to paleoparasitology with reports of ancient parasitic zoonosis. In 2014, the team described two calcified objects recovered from a fourth-century grave of a Roman adolescent in Amiens (northern France), which were identified as probable hydatid cysts [35]. Coprolites also represent key biological archives for reconstructing parasite transmission in ancient communities. Paleoparasitological investigation at the Kiasar archeological site (Caspian Sea littoral in northern Iran) dating from the Parthian era revealed the presence of *Dicrocoelium dendriticum* eggs in the grave of a young soldier and also showed the ancient occurrence of human dicrocoeliasis in this northern province of Iran, still known to be an endemic region for this parasite [5]. Interestingly, the discovery of *Fasciola hepatica* eggs in the paleofeces of an equid from the Chehrabad archaeological site, dated to the Sassanid period, prompted the authors to propose fascioliasis as a potential contributing factor to the historical decline of rare herbivores such as the Persian onagers in Iran [3]. Taken together, these findings highlight the complex interspecific interactions of parasites among human and animal hosts, processes that were markedly intensified by the advent of agriculture during the Neolithic period. Humans cannot remain insulated from the health of animals, agricultural systems, or the broader environment. Paleoparasitology offers a long-term perspective on how environmental changes, including climatic fluctuations and human migrations, have shaped parasite transmission dynamics over time. Such historical insights are crucial for anticipating how current and future environmental transformations may affect disease ecology. Paleoparasitology embodies the core principles of the “One Health” framework, as archaeological investigations integrate assessments of environmental conditions with both human and animal health.

## Toward an integrated multiparametric framework for “One Health”

Far from being a self-evident concept, “One Health” functions as an epistemic watchword [33], enabling assessment of the multiple dimensions of a public health problem. However, its practical implementation still has major limitations. A broader genuinely systemic perspective is therefore needed when applying the “One Health” framework to actions targeting parasitic or infectious diseases. The “Nine Planetary Boundaries” provide key biophysical indicators for defining such action plans [27, 49], delineating a safe and just operating space for humanity. Yet these parameters alone are insufficient and must be complemented by equally essential socio-economic considerations. Kate Raworth’s “doughnut” model offers such a perspective, proposing an alternative to traditional growth metrics by ensuring that essential human needs are met without exceeding planetary limits [41, 42].

Persistent gaps between scientific data and local perceptions highlight the limitations of unilateral, compartmentalized research approaches. More effective strategies require co-construction of knowledge that bridges environmental and social sciences. Applying the “One Health” lens allows evaluation and prioritization of interventions against parasitic diseases, while explicitly accounting for their ecological impacts. A multiparametric approach can thus be used to develop a synthesis table summarizing indicators, key actions, and their relevance for controlling cysticercosis [17, 21] within a One Health framework that integrates biogeophysical and socio-economic dimensions (Table 1). The proposed criteria emphasize human activities that shape parasite transmission dynamics, aiming to reduce trade-offs and enhance synergies across human, animal, and ecosystem health. These indicators can be applied both before and after interventions to guide sustainable and effective control strategies. This illustrates the fundamental inadequacy of single-parameter assessments, such as carbon footprint alone, for evaluating health interventions. Instead, multiparametric evaluation is necessary to design effective strategies against parasitic and vector-borne diseases. This approach is especially relevant given the growing global spread of non-homeothermic vectors (ticks, mosquitoes, and plant pests) driven by climate change and extensive transnational transport. Current chemical vector-control methods should therefore be assessed comparatively [2] using the parameters listed in Table 1. At equivalent cost, targeting vector agents globally is more efficient than addressing each pathogen they transmit separately.

**Table 1:** A “One Health” assessment model. Implementation of an action plan to combat porcine cysticercosis using different parameters deduced from the nine planetary limits defined by different authors and the expected socioeconomic consequences. This table is restricted to elements that are directly relevant to the control of cysticercosis. All planetary-boundary-type items (climate change, aerosols, ozone, ocean acidification, *etc.*) and generic governance concepts without a direct operational link have been removed (Boireau, 2024 International Academic Forum on Zoonoses Research, Jilin University, Changchun, PR China).

Domain	Relevant focus	Operational actions
*Biogeophysical Factors*		
Sanitation & Waste Management	Environmental contamination with human feces (primary driver of transmission)	Build and maintain latrines; prevent open defecation; prohibit use of untreated human feces as fertilizer; ensure safe disposal of waste.
Pig Management & Husbandry	Exposure of pigs to *Taenia solium* eggs	Keep pigs confined or tethered; separate pigs from human living areas; prevent access to latrines, waste dumps, and wastewater.
Land Use (Village Scale)	Human-pig environment interface	Organize village layouts that clearly separate housing, sanitation facilities, and pig-rearing areas; manage free-roaming livestock.
Food Safety & Meat Inspection	Transmission through infected pork	Promote local meat inspection; discourage informal slaughter without veterinary inspection; promote proper cooking of pork.
Freshwater Access & Hygiene	Fecal-oral transmission	Ensure access to safe drinking water; protect water sources from fecal contamination; promote handwashing with clean water.
*Socioeconomic and Public Health Factors*		
Human Health	Neurocysticercosis, epilepsy burden	Prioritize cysticercosis as a public health issue; integrate with epilepsy control programs.
Education & Awareness	Sustained behavior change	Implement community education on transmission routes, hygiene practices, pig confinement, and safe pork consumption.
Livelihoods & Economic Feasibility	Adoption and sustainability of control measures	Support affordable, small-scale pig production compatible with confinement and biosecurity; avoid costly industrial systems.
Housing & Village Organization	Environmental exposure and contamination	Improve housing design and spatial organization to separate humans, pigs, and sanitation facilities.
Child Protection	High vulnerability to infection	Reduce environmental contamination around homes; promote hygiene education and protective practices among children.
Local Governance & Community Involvement	Compliance, ownership, and long-term sustainability	Engage local authorities and community leaders in planning, implementation, monitoring, and enforcement of control measures.

During the COVID-19 pandemic, though much of the debate centered on whether the virus emerged from Chinese wet markets, the “One Health” framework remained significantly underutilized. Despite its potential, the French PREZODE initiative (https://prezode-initiative.org) focused primarily on infectiology and biological mechanisms, failing to sufficiently integrate the sociological and economic determinants of human behavior and ecosystemic disruption [39]. This limitation was already identified by Bim (2005), who underscored conceptual fallacies in early Gates Foundation “One Health” calls for proposals [4].

Moreover, the conventional schematic representation of “One Health” (Fig. 1) often portrays humans and animals as external to their ecosystem, which is an inaccurate depiction. A more realistic representation should place the ecosystem at the center of the framework [47]. Ecosystem health and biodiversity preservation remain critical yet neglected priorities. As Giraudoux emphasizes, while integrating human and animal health within ecosystems is logical and necessary in practice, the ecological dimension is still difficult to operationalize, in part because ecology is largely absent from healthcare training curricula [22–24].

This perspective echoes Virchow’s visionary statement that “*improving medicine can possibly prolong human life, but improving social conditions can achieve this result more quickly and more successfully*” [1]. It is also aligned with Amartya Sen’s fundamental question: “*Is health best promoted through the general process of economic growth… or is the advancement of health as a goal to be separated out from the process of economic growth seen on its own?*” [48]. Both historical and contemporary evidence consistently shows that addressing global public health challenges requires coordinated social, political, economic, and biomedical measures, a multidimensional integration that is still insufficiently reflected in many current “One Health” funding initiatives. “One Health” has evolved into a widely used term, yet a closer look reveals that professionals often still operate in silos: sociologists and economists, for instance, are rarely included. However, promising advancements toward cross-sector integration are emerging through the adoption of thinking systems and artificial intelligence [7, 38].

## Concluding remarks

Parasitology has made a major contribution to the “One Health” approach, which recognizes the interconnectedness of human, animal, and ecosystem health, particularly through studies on zoonotic parasites and vector-borne diseases. In practice, parasitologists have long applied One Medicine principles and occasionally those of “One Health” [45] to combat major zoonotic parasitoses, even before the term itself was coined, making them precursors of these concepts. Worldwide, many societies of parasitologists bring together biologists, ecologists, pharmacists, veterinarians, and physicians, reflecting the field’s inherently transdisciplinary nature. The life cycles of zoonotic parasites naturally span the three pillars of “One Health.” A simple examination of their diagrams vividly illustrates the rationale for this integrative approach to disease transmission. Even individuals with no formal education, in remote parts of the world, can observe such a diagram and identify the environmental, animal, plant, and behavioral factors involved in disease spread, and then explain these insights to others. As early as the 13th century, the Persian poet Saadi wrote in The Gulistan: “*Human beings are members of a whole, in creation of one essence and soul. If one member is afflicted with pain, other members will remain uneasy*.” This timeless wisdom resonates with the “One Health” concept: a change in balance in one domain inevitably affects the others. “One Health” is grounded on fundamental natural laws and requires a transdisciplinary approach, engaging not only environmental and health sciences, but also economics and the social sciences. This type of holistic perspective is indispensable for controlling major infectious diseases in humans, while safeguarding animal health and our fragile ecosystems. Importantly, parasitologists have embodied this approach for decades.

Nevertheless, our current perspective is inherently limited by its primary focus on the “One Medicine” component of the broader “One Health” framework. Genuine progress in health optimization demands a holistic perspective that fully integrates ecosystem health. Future work should explore not only how disease control measures influence ecosystems, but also how restoring and maintaining ecosystem health can, in turn, improve the well-being of both humans and animals. We encourage further exploration of these interconnected strategies in future articles.

## Appendix: Short biographies of parasitologists cited in the text

**Baltazard, Marcel** (1908–1971) was a French physician and biologist, former director of the Institut Pasteur of Iran and head of the epidemiology department at the Institut Pasteur in Paris.

**Cobbold, Thomas Spencer** (1828–1886) was a British parasitologist and physician known for his pioneering work in the study of parasitic diseases in humans and animals. He was one of the earliest specialists in helminthology and made significant contributions to understanding the relationship between animal and human parasitic infections, which aligns with early “One Health principles”. His numerous writings include Entozoa (1864); Human Parasites (1882); and Parasites of Meat and Prepared Flesh Food (1884).

**Golvan, Yves-Jean** (1928–2008) defined himself as a clinician, naturalist, and educator. A graduate in both medicine and science, he began his career in Paris under the influence of eminent scholars such as Robert Dollfus and Alain Chabaud. As an international expert on Acanthocephala, he was distinguished by his talents as a scientific illustrator and a rigorous systematician. After studying plague reservoirs in Kurdistan, he returned to France, where he founded the parasitology laboratory at Saint-Antoine University Hospital in Paris, and he was appointed as a professor. A pioneer of ecoepidemiology, he conducted foundational work on leishmaniasis in the Mediterranean and schistosomiasis in Guadeloupe. Furthermore, he published a list of fish genera that was used extensively by all fish parasitologists for decades (https://doi.org/10.1051/parasite/196237s6003).

**Harant, Hervé** (1901–1986) was a French parasitologist and zoologist. He was professor of natural history, parasitology, and tropical medicine at the Faculty of Medicine of Montpellier until 1971 and director of the Jardin des Plantes de Montpellier until 1976. JA Rioux succeeded him in the same functions.

**Hien, Tran Vinh** was a parasitologist trained in France and who worked with Jacques Lapierre at Cochin Hospital (Paris). In 1975, he moved to the Laboratory of Parasitology, Hospital for Tropical Diseases, Ho Chi Minh City, Vietnam where he was active until 2000 working on *Gnathostoma* and *Fasciola*.

**Osler, William** (1849–1919) was a Canadian physician and one of the founding figures of modern medicine. He was trained under Virchow and worked extensively on veterinary diseases, particularly zoonotic diseases like swine erysipelas.

**Rioux, Jean-Antoine** (1925–2017) was a French parasitologist, specializing in leishmaniasis. He coined the term ecoepidemiology for a discipline combining concepts of ecology and epidemiology to understand parasite transmission patterns and processes. He was a professor at the University of Montpellier and one of the founders of the “*Société Française de Parasitologie*” in 1962. Jean-Antoine Rioux was also a botanist and Director of the Jardin des Plantes de Montpellier, a historic botanical garden and arboretum.

**Ruffer, Marc Armand** (1859–1917) was a France-born British experimental pathologist and bacteriologist. He is considered a pioneer of modern paleopathology.

**Schwabe, Calvin W** (1927–2006) is often referred to as the “modern father of One Health”. He was a veterinary epidemiologist who reintroduced and formalized the concept in the 20th century. He advocated for the collaboration of human and veterinary medicine in combating zoonotic diseases as exemplified in his 1984 book “Veterinary medicine and human health”.

**Virchow, Rudolf** (1821–1902) was a German physician, pathologist, anthropologist, and politician, often called the “father of modern pathology.” Virchow coined the term zoonosis and emphasized the link between animal and human health. He believed that there should be no division between veterinary and human medicine. He developed the concept of cellular pathology, stating that “all cells come from other cells” (*omnis cellula e cellula*), which revolutionized medicine. Beyond science, he was also active in public health, politics, and social medicine, believing that medicine should serve society.

## References

[1] Ackerknecht E. 1953. Rudolf Virchow: doctor, statesman, anthropologist. Madison: University of Wisconsin Press.

[2] Angevin F Comité Scientifique du Haut Conseil des Biotechnologies. Avis en réponse à la saisine HCB du 12 octobre 2015 concernant l’utilisation de moustiques génétiquement modifiés dans le cadre de la lutte antivectorielle. https://hal.inrae.fr/hal-02789521v1; 2017 [accessed 15 June 2025].

[3] Askari Z, Mas-Coma S, Bouwman AS, Boenke N, Stöllner T, Aali A, Rezaiian M, Mowlavi G. 2018. *Fasciola hepatica* eggs in paleofaeces of the Persian onager *Equus hemionus onager*, a donkey from Chehrabad archaeological site, dating back to the Sassanid Empire (224–651 AD), in ancient Iran. Infection, Genetics, and Evolution, 62, 233–243. 10.1016/j.meegid.2018.04.028 29698771

[4] Bim AE. 2005. Gates’s grandest challenge: transcending technology as public health ideology. Lancet, 366, 514–519. 16084261 10.1016/S0140-6736(05)66479-3

[5] Bizhani N, Sharifi AM, Rokni MB, Dupouy Camet J, Rezaeian M, Fallah Kiapi M, Paknezhad N, Najafi F, Mowlavi G. 2017. *Dicrocoelium* egg identified in an ancient cemetery in Kiasar archeological site, Northern Iran, dated back 247 BC-224 AD. Iranian Journal of Public Health, 46(6), 792–795. 28828321 PMC5558072

[6] Bruschi F, Masetti M, Locci MT, Ciranni R, Fornaciari G. 2006. Short report: cysticercosis in an Egyptian mummy of the late Ptolemaic period. American Journal of Tropical Medicine and Hygiene, 74(4), 598–599. 16606991

[7] Burger PA. 2024. Integrating One Health into systems science. One Health, 18, 100701. 38468609 10.1016/j.onehlt.2024.100701 PMC10926283

[8] Cardiff RD, Ward JM, Barthold SW. 2008. “One medicine – one pathology”: Are veterinary and human pathology prepared? Laboratory Investigation, 88(1), 18–26. 18040269 10.1038/labinvest.3700695 PMC7099239

[9] Chandler AC. 1918. Animal Parasites and Human Disease. London: John Wiley & Sons.

[10] Delpech A. 1866. Les trichines et la trichinose chez l’homme et chez les animaux. Paris: Baillière et fils.

[11] Djurković-Djaković O, Dupouy-Camet J, Van der Giessen J, Dubey JP. 2019. Toxoplasmosis: Overview from a One Health perspective. Food and Waterborne Parasitology, 15, e00054. 32095624 10.1016/j.fawpar.2019.e00054 PMC7034049

[12] Dupouy-Camet J, Hueber T. 2021. La mission trichinose de 1866 d’Auguste Delpech et Jean Reynal en Allemagne: déjà une approche “Une seule santé’’. Bulletin de la Société Française d’Histoire de la Médecine et des Sciences Vétérinaires, 20, 77–94.

[13] Dupouy-Camet J, Murrell KD. 2007. FAO/WHO/WOAH Guidelines for the surveillance, management, prevention, and control of trichinellosis. Paris: Office international des epizooties.

[14] Eckert J, Gemmell MA, Soulsby EJL. 1981. FAO/UNEP/WHO guidelines for surveillance, prevention, and control of echinococcosis/hydatidosis. Geneva: World Health Organization.

[15] Estebanez J, Boireau P. 2022. One Health: A social science discussion of a global agenda. Parasite, 29, 17. 35315768 10.1051/parasite/2022014 PMC8939297

[16] FAO-WOAH-WHO Collaboration. Sharing responsibilities and coordinating global activities to address health risks at the animal-human-ecosystems interfaces A Tripartite Concept Note 2010. https://www.who.int/publications/m/item/the-fao-oie-who-collaboration; 2010 [accessed 15 June 2025].

[17] Garcia HH, O’Neal SE, Gilman RH, Cysticercosis Working Group in Peru. 2016. Elimination of *Taenia solium* Transmission in Peru. New England Journal of Medicine, 375(12), 1196–1197. 10.1056/NEJMc1609161 27653579

[18] Gebreyes WA, Dupouy-Camet J, Newport MJ, Oliveira CJ, Schlesinger LS, Saif YM, Kariuki S, Saif LJ, Saville W, Wittum T, Hoet A, Quessy S, Kazwala R, Tekola B, Shryock T, Bisesi M, Patchanee P, Boonmar S, King LJ. 2014. The global one health paradigm: challenges and opportunities for tackling infectious diseases at the human, animal, and environment interface in low-resource settings. PLoS Neglected Tropical Diseases, 8(11), e3257. 25393303 10.1371/journal.pntd.0003257 PMC4230840

[19] Gharbi M, Giraudoux P. 2024. Cystic echinococcosis (*Echinococcus granulosus sensu lato* infection) in Tunisia, a One Health perspective for a future control program. Parasite, 31, 30. 38874552 10.1051/parasite/2024029 PMC11177845

[20] Gibbs EP. 2014. The evolution of One Health: a decade of progress and challenges for the future. Veterinary Record, 174, 85–91. 24464377 10.1136/vr.g143

[21] Gilman RH, Gonzalez AE, Llanos-Zavalaga F, Tsang VC, Garcia HH, Cysticercosis Working Group in Peru. 2012. Prevention and control of *Taenia solium* taeniasis/cysticercosis in Peru. Pathogens and Global Health, 106, 312–318. 23265557 10.1179/2047773212Y.0000000045 PMC4005116

[22] Giraudoux P. 2022. Ecosystem health: What is the definition? Bulletin de l’Académie Vétérinaire de France, 175, 120–139.

[23] Giraudoux P, Besombes C, Bompangue D, Guégan JF, Mauny F, Morand S. 2022. One Health or “One Health washing”? An alternative to overcome now more than ever. CABI One Health. 10.1079/cabionehealth.2022.0006.

[24] Giraudoux P. 2023. One Health (Une seule santé) : concept nouveau en maturation ou vieille histoire? Bulletin de l’Académie Vétérinaire de France, 176, 224–236.

[25] Golvan YJ, Rioux JA. 1961. Écologie des mérions du Kurdistan Iranien. Relations avec l’épidémiologie de la Peste rurale (Enquête de l’Institut Pasteur de l’Iran. Rapport préliminaire). Annales de Parasitologie Humaine et Comparée, 36, 449–558. 13900030

[26] Golvan YJ. 1969. Eléments de Parasitologie médicale. Paris: Flammarion.

[27] Hansen J, Walker B, Liverman D, Richardson K, Crutzen P, Foley J. 2009. Planetary boundaries: exploring the safe operating space for humanity. Ecology and Society, 14, 32.

[28] Harant H. 1939. Parasitologie médicale. Paris: Maloine.

[29] Houin R, Léger N, Dupouy-Camet J, Bastien P, Luffau G. 2018). *In memoriam* Professor Jean-Antoine Rioux (1925–2017). Parasite, 25, 13.

[30] Kaplan B, Kahn LH, Monath TP, Woodall J. 2009. “One Health” and parasitology. Parasites & Vectors, 2(1), 36. 19674442 10.1186/1756-3305-2-36 PMC2729733

[31] Klob J. 1866. Bericht über die im Auftrage des hohen k. k. Staatsministeriums von den Professoren DDr. Müller und Klob zur Erforschung der Trichinenkrankheit unternommenen Reise nach Nord-Deutschland. Medizinische Jahrbücher, 6, 83–112.

[32] Liu M, Jin X, Boireau P. Biodiversity of food borne zoonotic parasites in China. Bulletin de l’Académie Vétérinaire de France, 178, 1–16.

[33] Michalon J. 2020. Accounting for One Health: Insights from the social sciences. Parasite, 27, 56. 33141659 10.1051/parasite/2020056 PMC7608981

[34] Morley NJ. 2025. Perceptions and misconceptions in visualizing parasite life cycles. Trends in Parasitology, 41, 344–347. 40157847 10.1016/j.pt.2025.03.003

[35] Mowlavi G, Kacki S, Dupouy-Camet J, Mobedi I, Makki M, Harandi MF, Naddaf SR. 2014. Probable hepatic capillariosis and hydatidosis in an adolescent from the late Roman period buried in Amiens (France). Parasite, 21, 9. 24572211 10.1051/parasite/2014010 PMC3936287

[36] Murrell KD, Dorny P. 2005. WHO/FAO/OIE guidelines for the surveillance, prevention, and control of taeniosis/cysticercosis. Paris: Office International des Epizooties.

[37] Noble ER, Noble GA. 1982. Parasitology, the biology of animal parasites (5th ed.). Philadelphia: Lea & Febiger.

[38] Parija SC, Poddar A. 2024. Artificial intelligence in parasitic disease control: A paradigm shift in health care. Tropical Parasitology, 14(1), 2–7. 38444798 10.4103/tp.tp_66_23 PMC10911181

[39] Peyre M, Vourc’h G, Lefrançois T, Martin-Prevel Y, Soussana JF, Roche B. 2021. PREZODE: preventing zoonotic disease emergence. Lancet, 397(10276), 792–793. 33640059 10.1016/S0140-6736(21)00265-8 PMC7946613

[40] Piekarski G. 1962. Medical parasitology in plates. Leverkusen: Bayer.

[41] Raworth KA. 2017. Doughnut for the Anthropocene: humanity’s compass in the 21st century. Lancet Planet Health, 1, e48–e49. 29851576 10.1016/S2542-5196(17)30028-1

[42] Raworth K. 2012. A safe and just space for humanity: Can we live within the doughnut? https://www.oxfam.org/sites/www.oxfam.org/files/dp-asafe-and-just-space-for-humanity-130212-en.pdf; [accessed 15 June 2025].

[43] Rioux JA, Houin R. 2009. Professeur Yves Golvan (1928–2008). Parasite, 16(3), 167–168.

[44] Robertson LJ, Utaaker KS, Goyal K, Sehgal R. 2014. Keeping parasitology under the One Health umbrella. Trends in Parasitology, 30, 369–372. 25022215 10.1016/j.pt.2014.06.002 PMC7128114

[45] Rohr JR, Sack A, Bakhoum S, Barrett CB, Lopez-Carr D, Chamberlin AJ, Civitello DJ, Diatta C, Doruska MJ, De Leo GA, Haggerty CJE, Jones IJ, Jouanard N, Lund AJ, Ly AT, Ndione RA, Remais JV, Riveau G, Schacht AM, Seck M, Senghor S, Sokolow SH, Wolfe C. 2023. A planetary health innovation for disease, food and water challenges in Africa. Nature, 619, 782–787. 37438520 10.1038/s41586-023-06313-z

[46] Rosolen SG, Dupouy-Camet J. 2023. Duel franco-prussien. Quand la revue scientifique d’Émile Alglave arbitrait les différents de Louis Pasteur et de Rudolf Virchow. Bulletin de l’Académie Vétérinaire de France, 176, 144–152.

[47] Selter F, Salloch S. 2023. Whose health and which health? Two theoretical flaws in the One Health paradigm. Bioethics, 37, 674–682. 37294266 10.1111/bioe.13192

[48] Sen A. Health in development. Bulletin of the World Health Organization, 77, 619–623. 10516784 PMC2557713

[49] Steffen W, Richardson K, Rockström J, Cornell SE, Fetzer I, Bennett EM, Biggs R, Carpenter SR, de Vries W, de Wit CA, Folke C, Gerten D, Heinke J, Mace GM, Persson LM, Ramanathan V, Reyers B, Sörlin S. 2015. Sustainability. Planetary boundaries: guiding human development on a changing planet. Science, 347, 1259855. 25592418 10.1126/science.1259855

[50] Virchow R. 1860. Note sur *Trichinella spiralis*. Comptes Rendus Hebdomadaires de l’Académie des Sciences, 51, 13–16.

[51] Yang G, Zhou X. 2023. One Health concept: viewed by Chinese traditional philosophy from a millennial history. CABI One Health. 10.1079/cabionehealth.2023.0010; [accessed 15 June 2025].

[52] Zenker FA. 1860. Über die Trichinen-Krankheit des Menschen. Virchow’s Archiv für pathologische Anatomie und Physiologie und für klinische Medicin, 18, 561–572.

[53] Zhang G, Qiu Y, Boireau P, Zhang Y, Ma X, Jiang H, Xin T, Zhang M, Tadesse Z, Wani NA, Song J, Ding J. 2024. Modern agriculture and One Health. Infectious Diseases of Poverty, 13, 74. 39385259 10.1186/s40249-024-01240-1 PMC11466017

